# Community-Led Cancer Action Councils in Queens, New York: Process Evaluation of an Innovative Partnership With the Queens Library System

**DOI:** 10.5888/pcd11.130176

**Published:** 2014-02-06

**Authors:** Upal Basu Roy, Tamara Michel, Alison Carpenter, David W. Lounsbury, Eilleen Sabino, Alexis Jurow Stevenson, Sarah Combs, Jasmine Jacobs, Deborah Padgett, Bruce D. Rapkin

**Affiliations:** Author Affiliations: Upal Basu Roy, New York University School of Medicine, New York, New York; Tamara Michel, Jasmine Jacobs, Queens Library, Jamaica, New York; Alison Carpenter, Eilleen E. Sabino, Alexis Jurow Stevenson, Sarah Combs, Bruce D. Rapkin, Albert Einstein College of Medicine, Bronx, New York; Deborah Padgett, Silver School of Social Work, New York University, New York, New York.

## Abstract

**Introduction:**

Community-based participatory research (CBPR) has great potential to address cancer disparities, particularly in racially and ethnically diverse and underserved neighborhoods. The objective of this study was to conduct a process evaluation of an innovative academic–community partnership, Queens Library HealthLink, which aimed to reduce cancer disparities through neighborhood groups (Cancer Action Councils) that convened in public libraries in Queens, New York.

**Methods:**

We used a mixed-methods approach to conduct 69 telephone survey interviews and 4 focus groups (15 participants) with Cancer Action Council members. We used 4 performance criteria to inform data collection: action or attention to sustainability, library support for the council, social cohesion and group leadership, and activity level. Focus group transcripts were independently coded and cross-checked for consensus until saturation was achieved.

**Results:**

Members reported benefits and barriers to participation. Thirty-three original focus group transcript codes were organized into 8 main themes related to member experiences: 1) library as a needed resource, 2) library as a reputable and nondenominational institution, 3) value of library staff, 4) need for a HealthLink specialist, 5) generation of ideas and coordination of tasks, 6) participation challenges, 7) use of community connections, and 8) collaboration for sustainability.

**Conclusion:**

In response to the process evaluation, Cancer Action Council members and HealthLink staff incorporated member suggestions to improve council sustainability. The councils merged to increase intercouncil collaboration, and institutional changes were made in funding to sustain a HealthLink specialist beyond the grant period.

## Introduction

The Queens Library HealthLink program is a community-based participatory research (CBPR) partnership among Queens Library, Albert Einstein College of Medicine, the American Cancer Society, and Queens Cancer Center of Queens Hospital in New York City. CBPR provides a means for tackling complex and challenging public health issues at the community level (1–3). In 2006, HealthLink received a 5-year grant from the National Cancer Institute to decrease rates of late-stage cancer detection and reduce overall cancer disparities in 20 medically underserved neighborhoods in Queens. Through HealthLink, the public libraries were an accessible source of health information and a trusted platform for community organizing; in addition, they served as sites for meetings, events, and information dissemination.

Neighborhoods in Queens vary by cancer mortality among residents and by demographic factors such as country of birth, race, and ethnicity of residents. Late-stage detection rates in Queens for breast and prostate cancers are 6 times the national rates (4); some locations have rates as high as 2.7 times greater than the rate for New York City as a whole (5).

Neighborhoods were selected for participation in the HealthLink program according to the following factors: the percentage of residents living below federal poverty guidelines (only neighborhoods in which more than 11% of residents lived below the guidelines were considered), the percentage of foreign-born residents, the percentage of African American residents, the degree of racial and ethnic diversity (roughly equal proportions of white, African American, Hispanic, and Asian), and the extent of the cancer burden based on New York State cancer registry data and New York City health department statistics (6,7). These criteria reflect high rates of late-stage cancer diagnoses, low rates of cancer screening availability and use, and general vulnerability to poor health outcomes.

A key element of the HealthLink intervention was the establishment of Cancer Action Councils composed of community volunteers. Council members were recruited via letters, telephone calls, and e-mails to local community- and faith-based organizations. Members were also encouraged to invite other local stakeholders to council meetings and events. With library support, council members planned and implemented cancer screenings, tours of health care facilities, and health-related workshops; created informational materials; and organized programs combining entertainment or crafting (eg, quilting) with cancer education. We recruited people who lived or worked in each selected community and were knowledgeable about their own community’s needs. Most members represented local organizations or Queens Library or participated because they had personal experience with cancer. Councils served as inter-organizational links through which members could work and share resources, thereby making the programming more relevant to local cultures (8,9).

The HealthLink model included 2 grant-funded HealthLink specialists (trained in public health) for 1 year to provide guidance to the councils, with the goal of becoming self-sustaining after that period. Following the basic tenets of CBPR, the councils planned and conducted their own events, ensuring that they had control over programs, which they tailored to the unique needs of their communities (10,11). Programs were held in various locations in and outside the libraries and were designed to reach various age and language groups. The HealthLink project proceeded in phases whereby 2 councils were established every 4 months starting in October 2007 and ending after 20 councils were convened.

The objective of this study was to assess the implementation of HealthLink, including the level of participation among the councils, the extent of community reach, and prospects for sustainability. We addressed the following research questions: What were the incentives and obstacles to participation? How satisfied were council members with their experience? How can the councils provide for sustainability?

## Methods

We used mixed methods to assess HealthLink implementation. Mixed methods permitted evaluation of HealthLink from different vantage points. We administered a semistructured telephone survey in 2010 and conducted focus group interviews among council members in 2011. The academic partners’ human subjects committees approved all study protocols.

### Member survey

To be eligible for participation in the survey, a council member must have belonged to a council that existed for at least 6 months and must have attended at least 1 meeting. Our reason for including minimally active members was to ensure that we obtained feedback on factors that might have discouraged participation. The sample included members from 18 of 20 councils; 69 of 103 current or former council members completed the survey, a 61% response rate.

The survey items, some of which were adapted from the CBPR literature (12–15), examined demographic characteristics and aspects of the council members’ experience. The survey asked whether the member represented an organization and (if so) which type, member’s expectations for the council, council activity and goal fulfillment, future goals, input in decision making, benefits and drawbacks of participation, and satisfaction with participation. Survey administration took about 25 minutes per participant. Survey data were entered into SPSS software (IBM Corporation, Armonk, New York) for analyses.

### Focus groups

Using a maximum variation sampling approach (16), we organized 4 focus groups, 2 consisting of members from high-performing councils and 2 consisting of members from low-performing councils. We sought to engage 4 to 5 council members for each focus group. Members from middle-performing councils were not included because we sought to identify only the highest and lowest limits of performance.

The first step in recruiting focus group participants was to determine the high- and low-performing councils from which to recruit. We considered only councils that had at least 2 meetings in the previous 6 months. Of the 20 councils, 16 met this criterion. Two HealthLink specialists and the project coordinator independently rated these 16 councils as low, middle, or high performing, based on 4 criteria: action or attention to sustainability, neighborhood library support, social cohesion and group leadership, and activity level. Each rater awarded 1 point to low-performing, 2 points to middle-performing, and 3 points to high-performing councils. Ten councils were categorized as high performing or low performing, and 6 were rated as middle performing.

The focus group interview guide consisted of questions related to the same 4 performance criteria that were used to rate the performance of the councils. The following are examples of questions: How has the Queens Library contributed to the work of your council? How is the work of organizing an event carried out? What would your council need to keep functioning?

A research team member who had experience in leading focus groups and had minimal contact with council members facilitated the sessions. Each focus group lasted approximately 90 minutes and was audio recorded and transcribed verbatim. All participants were given $20 and a $5 MetroCard for round-trip fare on public transit. Nine members from 2 high-performing councils participated in 2 focus groups (4 in one group; 5 in the other), and 6 members from 2 low-performing councils participated in 2 focus groups (2 in one group; 4 in the other).

Analysis of the focus group transcripts involved repeated independent readings by 2 members of the research team followed by meetings to discuss emergent codes and agree on a master codebook of 33 codes. Using the codebook, the 2 researchers independently coded the transcripts. NVivo software (QSR International, Victoria, Australia) was used to enter the codes and to derive coded excerpts for thematic analysis. Common themes were identified through discussion and consensus; memoing was used throughout to document decisions and to define themes and their grounding in the data (17).

## Results

### Survey results

Of the 69 survey participants, 77% were women, 48% were African American, and 68% represented a local organization (Table). Participants reported that they agree or strongly agree that community interests are well represented in council projects (78%) and that council members have a voice in the development of programs (97%). Participants (84%) also reported a sense of ownership in their council’s accomplishments.

**Table:** Characteristics of Participants in Cancer Action Coalitions, Queens, New York, 2010 and 2011

Characteristic	n (%)
**Member Survey^a^ (N = 69)**	
**Sex**	
Female	53 (77)
Male	16 (23)
**Race/ethnicity**	
White	17 (25)
African American	33 (48)
Asian	7 (10)
American Indian or Alaska Native	1 (1)
Other	11 (16)
**Place of residence**	
Lives in the community served by the Cancer Action Council	35 (51)
Does not live in the community served by the Cancer Action Council	34 (49)
**Agency representation**	
Participates in Cancer Action Council on behalf of local organization	47 (68)
Independent volunteer	22 (32)
**Agency type represented^b^**	
Community	19 (40)
Library	13 (28)
Health care	9 (19)
Religious	2 (4)
Government	3 (6)
Nongovernment	1 (2)
**Role or position at agency^b^**	
Executive/officer	9 (19)
Manager/administrator	17 (36)
Faculty/teacher	4 (9)
Division director/department head	6 (13)
Clinician/direct service provider	4 (9)
Other	7 (15)
**Focus Group (N = 15)**	
**Sex**	
Female	10 (67)
Male	5 (33)
**Race/ethnicity**	
White	4 (27)
African American	9 (60)
Asian	2 (13)

a 69 of 103 current or former council members completed the survey, a 61% response rate.

b Percentages are based on the number of participants (n = 47) affiliated with an organization; 22 participants were independent volunteers.

When asked about the major challenges facing their councils, the greatest percentage of participants (36%) reported challenges in sustaining or increasing participation. Participants also voiced concern about securing future funding (23%). Another major challenge was reaching certain populations, such as non-English speakers, with outreach efforts (17%).

Survey participants also reported benefits of participation. The top 5 responses were 1) acquired useful knowledge about programs, services, or people in the community (97%), 2) developed valuable relationships (94%), 3) increased ability to contribute to communities (94%), 4) made a greater impact than they could have on their own (91%), and 5) enhanced ability to address an important issue (88%). When asked to name major accomplishments, participants most commonly reported the planning and hosting of events (39%), cancer screenings (23%), and health fairs (23%).

### Focus group results

Of the 15 focus group participants, 10 were women and 9 were African American (Table). Qualitative analyses yielded 8 themes: 1) library as a needed resource, 2) library as a reputable and nondenominational institution, 3) value of library staff, 4) need for a HealthLink specialist, 5) generation of ideas and coordination of tasks, 6) participation challenges, 7) use of community connections, and 8) collaboration for sustainability.

**Library as a needed resource:** Participants noted the public library as a valuable resource. One member from Focus Group 4 stated, “Now we know where I could go. You know the library has many books . . . they have a resource place, I can go on the website . . . and get me a link, you know, different things like that.” Participants reported using the library’s marketing department to create and distribute educational and promotional flyers and to share information via the library’s website. One member explained during Focus Group 2, “[Printed materials are] distributed throughout the [library] system, so that every branch that you go to, you will find something related to cancer.”

Participants also acknowledged libraries as community spaces where they could reach larger audiences already gathered for other library programs. One member stated during Focus Group 2, “The last time we scheduled a health fair, we had the business starter program. We had a lot of attendees for that, probably like 75, and as they were leaving the auditorium, they walked right into the area where we’re keeping the health fair.”

**Library as a reputable and neutral institution:** Participants agreed that the library is an ideal venue for community organizing, especially in an area as diverse as Queens. One member explained during Focus Group 2, “Everybody comes to the library. I don’t care what religion you are, status you are. . . .The library is for everyone.”

**Value of library staff:** Participants stressed the relationship between council effectiveness and the presence of a library staff member committed to the HealthLink mission. One participant stated during Focus Group 2, “I think you’re so successful . . . because [name] is the librarian . . . she has incorporated her Cancer Action Council into her job duties.” During Focus Group 3, another stated, “[There are] certain communities that are more willing, or where the turnout is high, because, again, I think it’s effort that’s coming from . . . the librarian.”

**Need for a HealthLink specialist: **Participants asserted that the HealthLink specialists were essential partners in community outreach. Most members were uncomfortable with the temporary role of the HealthLink specialists (designed to last 1 year and then become locally sustained). For example, one participant explained during Focus Group 3 that “[The HealthLink specialists] are very proactive getting things done. It makes it easy on us. We don’t have to exert all this effort because we still have our own jobs to do, in addition to collaborating with the Cancer Action [Council]. So they make things pretty much easy for us.”

**Generation of ideas and coordination of tasks:** Participants reported that they used monthly meetings primarily to generate new ideas, especially for large-scale projects, and to assign responsibilities; coordination of tasks was also done by telephone or e-mail. One member from Focus Group 1 explained,

Someone may come up with an idea and then from that idea something else will branch out. But at my council everyone has something to say. . . . I had mentioned having a health miniseries here at the library, and that each member would provide a workshop. Because we don’t have the funding to hire someone to come in and provide the workshop, we would have to do the research and then either find a presenter or give the presentation ourselves. My [colleague] . . . came up with an even better idea: she said well, let’s just reach out to the local hospital.

Another member from Focus Group 1 discussed how the council determines whether or not an event is possible to organize:

I personally have said why don’t we do this type of event, and then I would say let’s just go around the table and see what we can all provide. And if there’s 100% participation . . . then it’s something we can do. But if it’s just 1 person or 2 people that have resources, and the rest of us really don’t know how to do outreach for this particular event, then it may not be the best event for us for now.

**Participation challenges:** Participants reported that although some council events were well attended, it was hard to recruit members from among those who came. For example, 1 participant from Focus Group 1 commented on the challenges of recruiting*:* “They would come to your events but we want them also to come and . . . be an active member of the council, and that’s where it’s been hard trying to get people to actually stick with us.”

**Use of community connections:** Use of their professional connections and involvement within the community were 2 ways in which council members could carry out events and increase participation within their councils. One member from Focus Group 1 commented, “We’re thinking [about what] we’re each expected to bring to the table. Who can we involve through our connections? Who can make this event happen?”

Another participant mentioned during Focus Group 1 that her membership in a local church increased participation. “We were having our [council] meeting at the church . . . and the pastor was there. What’s so important is . . . if he [pastor] says it from the church, from the pulpit, they come, and I think that’s why it was so successful.”

**Collaboration for sustainability.** Participants noted that collaboration with other councils and local community organizations is a vital part of sustainability. Some members suggested merging with other councils as a means of becoming more sustainable. One from Focus Group 1 stated, “We’re trying to get the surrounding communities and other [Cancer Action Councils] to work with us. I think we may need to merge with a neighboring group . . . even the ones that are not right next to us may all want to collaborate and do something together.” Another participant in Focus Group 1 described the importance of strategic partnerships: “One of the things that I’ve been thinking about for sustainability is Head Start. . . . Why? Because Head Start needs partnerships.”

### Integrating the quantitative and qualitative results

Quantitative and qualitative findings showed consistency in satisfaction with the project and council activities, benefits from participation, and concerns about sustainability. Although we found no areas of disagreement between the 2 sets of findings, the focus groups provided in-depth and spontaneous information that complemented and deepened the survey results, including information on the difficulties of recruiting new council members, the important role of the HealthLink specialists, the benefits of library involvement, creative strategies for partnering with other community organizations, and suggestions for enhancing sustainability.

## Discussion

We observed both the benefits and challenges of academic–community partnerships in our study. Although we found no disagreements over goals or activities between council members and academic partners (the project emphasized council control), we found barriers to program implementation, including recruitment and retention of council members and uneven performance among councils. Of serious concern was the issue of sustainability and ways to maintain key elements of the HealthLink model. Without adequate funding or local support, for example, the HealthLink specialist role is in jeopardy. Similarly, outreach and educational activities require infrastructure support beyond volunteer time and effort. On a positive note, the study participants emphasized the pivotal role of the library, a venue that was accessible, cost-effective, and aligned with the library system’s goals of information access. Indeed, library staff involved in HealthLink noted the increased standing of the library as a center for health promotion in the community.

The survey had a 61% response rate, but despite efforts to encourage attendance, we had a small number of focus group participants. As a study strength, strategies for rigor used in qualitative inquiry (17) included independent ratings and coding, auditing or memoing all analytic decisions, and peer debriefing.

Through this process evaluation, members identified new ways to improve the functioning and sustainability of their councils. For example, the need to foster greater collaboration and pool resources led to a joint decision by the councils to merge into 4 regional councils. This merge created larger-scale programs that can reach a broader range of community members through shared resources within and across neighborhoods. Additionally, to increase participation, the newly formed regional councils altered the frequency of their local meetings to account for member availability and productivity. Some councils chose to continue holding local meetings and planning programs tailored to the needs of their immediate community.

To ensure that council members could continue their work after the initial funding period, the Queens Library Foundation secured funds from a private foundation and retained 1 full-time HealthLink specialist. The HealthLink staff also held capacity-building workshops for council members to make them more comfortable with performing some of the duties typically carried out by HealthLink specialists.

Programs resulting from CBPR benefit from being located in a neutral, welcoming location such as a public library. Our study highlighted obstacles to implementation but also demonstrated successes and new directions for sustainability that were subsequently enacted. This feedback loop enabling program improvement was critical to the program’s continuation beyond the funding period.

Several council members identified themselves as cancer survivors. Much is known about programs developed for cancer survivors, but little research has examined how survivors may contribute to cancer control in local communities. As the population of cancer survivors approaches more than 15 million in the United States (18), cancer survivors are an untapped resource in cancer prevention and support.

Finally, we call attention to a key innovative aspect of this project: its use of public libraries to create a bridge between academic and community partners. Although HealthLink is a work in progress, its reliance on a trusted neighborhood institution increased prospects for sustainability. By developing relationships with council members through the local libraries, HealthLink academic and medical partners learned about local community needs and how to most effectively reach people in their neighborhoods, thus allowing the partners to further their missions.
